# Three-dimensional modeling of DCIR and identification of new drugs blocking HIV-1 attachment and propagation

**DOI:** 10.1186/1742-4690-9-S1-P9

**Published:** 2012-05-25

**Authors:** Caroline Gilbert, Arezki Azzi, Alexandra A Lambert, Sheng-Xiang Lin, Geneviève Allaire, Karianne P St-Gelais, Michel J Tremblay

**Affiliations:** 1 Laval University, Québec, Canada

## Introduction

The HIV-1 pandemic continues to expand while no effective vaccine is yet available. Finding new therapeutic targets and drugs is therefore crucial. We have previously shown that the dendritic cell immunoreceptor (DCIR), a C-type lectin receptor expressed in dendritic cells (DCs), acts as an attachment factor for HIV-1 to DCs and contributes to HIV-1 transmission to CD4+ T lymphocytes (CD4TL). Directly involved in HIV-1 infection, DCIR is expressed in apoptotic or infected CD4TL and promotes trans-infection to bystander cells. The aim of the present study is to characterize the extracellular domain of DCIR and to test chemical inhibitors of HIV-1 attachment thereto.

## Results

We present the first three-dimensional model of DCIR structure. Based on this structure, several inhibitors were selected to target viral interaction with the carbohydrate recognition domain and the EPS motif. Preliminary screening using Raji-CD4-DCIR cells identified two inhibitors that decreased HIV-1 attachment and propagation. These inhibitors did not affect the proliferation of peripheral blood mononuclear cells.

## Conclusions

The results of this study thus suggest structures for novel molecules capable of blocking HIV-1 transmission by DCs and CD4TL.

